# Delivery of mRNA encoding an anti‐tau monoclonal antibody and engineered scFv intrabody results in functional antibody expression in neurons

**DOI:** 10.1002/alz.087103

**Published:** 2025-01-03

**Authors:** Patricia Wongsodirdjo, Alayna C Caruso, Alicia Yong, Madeleine Lester, Laura J Vella, Ya Hui Hung, Rebecca M Nisbet

**Affiliations:** ^1^ The Florey Institute of Neuroscience and Mental Health, Melbourne, VIC Australia; ^2^ The University of Melbourne, Melbourne, VIC Australia

## Abstract

**Background:**

Monoclonal antibodies have emerged as a leading therapeutic agent for the treatment of disease, including Alzheimer’s disease. Such antibodies, however, are expensive and timely to produce and require frequent dosing regimens to ensure disease‐modifying effects. Synthetic *in vitro*‐transcribed mRNA encoding antibodies presents a promising alternative to conventional passive immunotherapy and overcomes the need to generate recombinant antibodies.

**Method:**

I*n vitro*‐transcribed (IVT) mRNA encoding a tau specific antibody as a full‐sized IgG and as a single chain variable fragment (scFv) was synthesised. Human neuroblastoma SH‐SY5Y cells and mouse primary hippocampal neurons were transfected with the IVT mRNA and antibody translation and ability to bind tau was assessed.

**Result:**

Transfection of mammalian cells with IVT synthesised mRNA results in the translation of a secreted tau‐specific IgG and an intracellular scFv intrabody. Importantly, both antibody formats engage tau, with the intrabody engaging tau directly within the cell cytoplasm.

**Conclusion:**

IVT synthesised mRNA can be used to generate both functional full‐length IgG antibodies and scFv intrabodies targeting tau. Our study highlights the utility of mRNA as an inexpensive and versatile delivery platform for antibody therapeutics.

